# Biocontrol Approaches against *Escherichia coli* O157:H7 in Foods

**DOI:** 10.3390/foods11050756

**Published:** 2022-03-05

**Authors:** Pradeep Puligundla, Seokwon Lim

**Affiliations:** Department of Food Science & Biotechnology, Gachon University, 1342 Seongnam-daero, Sujeong-gu, Seongnam-si 13120, Korea; puli@gachon.ac.kr

**Keywords:** biocontrol, *Escherichia coli* O157:H7, food, bacteriophage, bacteriocin, endolysin, probiotic, hurdle technology

## Abstract

Shiga-toxin-producing *Escherichia coli* O157:H7 is a well-known water- and food-borne zoonotic pathogen that can cause gastroenteritis in humans. It threatens the health of millions of people each year; several outbreaks of *E. coli* O157:H7 infections have been linked to the consumption of contaminated plant foods (e.g., lettuce, spinach, tomato, and fresh fruits) and beef-based products. To control *E. coli* O157:H7 in foods, several physical (e.g., irradiation, pasteurization, pulsed electric field, and high-pressure processing) and chemical (e.g., using peroxyacetic acid; chlorine dioxide; sodium hypochlorite; and organic acids, such as acetic, lactic, and citric) methods have been widely used. Although the methods are quite effective, they are not applicable to all foods and carry intrinsic disadvantages (alteration of sensory properties, toxicity, etc.). Therefore, the development of safe and effective alternative methods has gained increased attention recently. Biocontrol agents, including bacteriophages, probiotics, antagonistic bacteria, plant-derived natural compounds, bacteriocins, endolysins, and enzymes, are rapidly emerging as effective, selective, relatively safe for human consumption, and environmentally friendly alternatives. This paper summarizes advances in the application of biocontrol agents for *E. coli* O157:H7 control in foods.

## 1. Introduction

Shiga-toxigenic *Escherichia coli* (STEC), also known as verotoxigenic (VTEC) or enterohemorragic (EHEC) *E. coli*, comprise an important group of bacterial pathogens. STEC is a common cause of foodborne infections, accounting for more than 265,000 illnesses per year in the United States [1]. *E. coli* O157:H7 is the most notable STEC serotype with a low infectious dose (50–100 colony-forming units (CFU/g or mL)) and it has been shown to cause a variety of human diseases, such as mild diarrhea, hemorrhagic colitis, hemolytic uremic syndrome, and thrombotic thrombocytopenic purpura [2]. The pathogenicity of STEC is due to Shiga toxins (encoded by *stx*1 or *stx*2 genes) production, together with additional acquired virulence features [3].

The major routes of transmission of *E. coli* O157:H7 throughout the world are poultry, livestock, and their meat products [4], and most outbreaks in the past have been associated with the consumption of undercooked ground beef or drinking raw milk (less frequently) [5,6]. Al-kuzayi and Al-Sahlany [7] showed a high incidence of *E. coli* O157:H7 in dairy products (especially white soft cheese and braided cheese samples) collected from markets in Basra city. Food items of non-bovine origin, such as water, vegetables (e.g., baby spinach, shredded lettuce), apple cider, and cantaloupe were found to be linked to *E. coli* O157:H7 infection [6,8].

*E. coli* O157:H7 is fairly resistant to various stresses; the bacteria can survive for a certain period in soil and manure (outside of a host), potentially leading to the contamination of vegetables and fruits [9]. However, its survival in the environment can be affected by factors including temperature, dehydration, indigenous microbial communities, moisture, and aerobic conditions. It was shown that *E. coli* O157:H7 can survive and/or grow in a variety of minimally processed vegetables and fruits, including shredded carrot, sliced cucumber, shredded lettuce, soybean sprouts, packaged fresh-cut salad, dry coleslaw mix, peaches, and honeydew melon, at abuse temperatures [10]. In addition, the ability of *E. coli* O157:H7 to survive during food processing and subsequent recovery and growth during refrigerated storage was demonstrated experimentally [11,12]. The robust survival exhibited by *E. coli* O157:H7 under most conditions emphasizes the significance of having appropriate sanitation and disinfection methods to deal with it [9]. 

The application of heat has been an important technology to kill this pathogen; the pasteurization of milk at 72 °C can eliminate these pathogens [9]. However, it is widely known that thermal processing may negatively affect the nutritional and functional properties of foods [13]. As alternatives to thermal processing, several studies have reported the potential of non-thermal treatments, such as irradiation technologies (which typically use ionizing radiation, such as γ-rays, low-dose electron beam, and X-rays), UV irradiation (e.g., UV-C and pulsed UV light), high-pressure processing (HPP), and pulsed electric fields (PEFs) to eliminate *E. coli* O157:H7 in foods [9,14,15,16,17]. These non-thermal technologies are not without disadvantages, such as high capital cost, regulatory hurdles, and being inappropriate for some foods. On the other hand, relatively inexpensive chemical sanitizers, such as peroxyacetic acid, chlorine dioxide, sodium hypochlorite, acidified sodium chlorite, organic acids (e.g., acetic, lactic, and citric), and aqueous ozone have been popularly used to reduce the prevalence of *E. coli* O157:H7 and other food-borne pathogens on raw meat products [18]. The relative antimicrobial effectiveness of these compounds was determined as being organic acids > peroxyacetic acid > chlorinated compounds > aqueous ozone. For the sanitization of fresh-cut produce, chlorine-based sanitizers, such as sodium hypochlorite, are commonly used owing to their cost-effectiveness and ease of implementation. Gonzalez et al. [19] showed that, among the tested sanitizers (200 ppm chlorine, 1000 ppm acidified sodium chlorite, 80 ppm peroxyacetic acid-based sanitizer, citric acid-based sanitizer), acidified sodium chlorite was the most effective at reducing the levels of *E. coli* O157:H7 (5.25 log reduction) on fresh-cut carrot shreds. However, the efficacy of chlorine on pathogens is limited and it loses its activity quickly upon contact with organic matter, contact with metals, increased temperature, and exposure to light [19]. In addition, water chlorination can lead to the generation of undesirable by-products, such as trihalomethanes, and therefore, European countries have prohibited the use of chlorine-based sanitizers [20]. 

Sodium benzoate and potassium sorbate are potent and highly useful agents for food and beverage preservation. Malic acid, sodium benzoate, potassium sorbate, and their combinations were found to significantly decrease the D (decimal reduction time) value of *E. coli* O157:H7 in apple cider [21,22]. However, there are safety concerns. For instance, benzoate can react with ascorbic acid in drinks to produce the carcinogen benzene [23]. These disadvantages necessitate the development of safe, effective, and acceptable antimicrobial agents with a limited ecological footprint. In recent years, biocontrol approaches are becoming increasingly attractive for the control of food-borne pathogens because of ever-increasing antimicrobial resistance, as well as consumer awareness of the health risks of chemical food additives and preservatives [24].

Biocontrol agents, such as bioprotective microorganisms (bacteriophages, probiotics, and other antagonistic bacteria), plant-derived natural compounds, bacteriocins, and endolysins, have been used against *E. coli* O157:H7. Many studies reported success with these agents. The primary criteria for choosing a biocontrol agent include high antagonistic activity against the pathogen, safe for human consumption, and it should not alter the nutritional and organoleptic properties of food products [24,25]. In the present study, up-to-date developments in biocontrol approaches against *E. coli* O157:H7 were systematically reviewed.

## 2. Bacteriophages

Phages constitute the largest group of viruses, and they utilize species in the Archaea and Bacteria domains as hosts [26]. Phages are self-replicating entities that can multiply by taking control of their host’s DNA replication and protein synthesis machinery. Phages can be categorized into lytic (virulent) or lysogenic (temperate) based on their infection life cycle. Phage-mediated biocontrol or phage therapy is the killing of pathogenic or nuisance bacteria via the application of bacterial strain-specific or species-specific phages [27]. Phages have several advantages compared with conventional antibacterial agents, including highly host-specific, rapid lysis of bacteria, and being non-infectious and non-toxic (except certain cell lysis may release endotoxins) to humans [28]. Kudva et al. [29] isolated bacteriophages specific to *E. coli* O157 antigen and found that a mixture of KH1, KH4, and KH5 phages was able to lyse *E. coli* O157 from cultures. Critical factors for rapid lysis included simultaneous infection with the three phages, a high multiplicity of infection (MOI), aeration, and incubation at 37 °C. In a study, the application of lytic phages (e11/2 and pp01) and a cocktail of virulent phages (e11/2, e4/1c, and pp01) against *E. coli* O157:H7 had resulted in a 5-log-unit reduction of pathogen viable counts at temperatures of 30 or 37 °C, where active growth of the organism was observed; however, the lytic ability was substantially decreased at 12 °C due to the absence of culture growth, indicating that the metabolic processes associated with host cell growth are necessary for the replication of *E. coli* phage [2]. Even though the emergence of bacteriophage-insensitive mutants (BIMs) was noted following the application, BIMs subsequently reverted to phage sensitivity. The phage vB_EcoS_AKFV33 (AKFV33) was shown to be highly virulent to various phage types of STEC O157 strains [30]. 

### 2.1. Phages for E. coli O157:H7 Control in Foods

Phage biocontrol strategies for food preservation have significant advantages, such as being highly discriminatory, cost-effective, self-perpetuating, and natural, and they do not alter food composition and sensorial properties [28,31]. However, there are some drawbacks as well, which include the requirement for threshold numbers of the bacterial targets, a limited host range, the potential for the transduction of undesirable characteristics from one bacterial strain to another, and phage-resistant mutants [31]. The potential of the phage FAHEc1 for *E. coli* O157:H7 reduction on sliced beef pieces was shown [32]. Compared to phage-free controls, significant reductions in *E. coli* O157:H7 (>4 log_10_ CFU/cm^2^) on raw and cooked beef pieces were achieved with the phage FAHEc1 at 5 °C and 24 °C [33]. Their results showed that a minimum phage concentration of about 10^4^–10^5^ plaque-forming units (PFU)/cm^2^ was required for the occurrence of reductions. They also showed that a concentration of 8 log_10_ PFU/cm^2^ was required to achieve a reduction of 4 log_10_ CFU/cm^2^. A virulent bacteriophage specific for *E. coli* O157:H7, namely, M8AEC16, was used as a biocontrol agent in Turkish raw meatball [34]. They showed a significant decrease of viable *E. coli* O157:H7 counts with increasing phage concentration; a significant effect of MOI, but neither time nor storage conditions, was observed for bacterial counts. In another study, the phage M8AEC16 was shown to be effective for decontaminating *E. coli* O157:H7 in ready-to-eat (RTE) salads [35]. The efficacy of e11/2 and e4/1c phages to control *E. coli* O157:H7 in foods was demonstrated by Coffey et al. [36]. Both the phages considerably reduced the viable counts of *E. coli* O157:H7 upon exposure under varied conditions of pH values (from >4 to 9), water activity values (0.87 or 0.91 to 1.00), temperatures (from 4 °C to 37 °C), and NaCl concentrations (1% to 2.5%). Coliphage ECP4 belonging to the *Siphoviridae* family was used for the biocontrol of *E. coli* O157:H7 [37]. Viable counts of the pathogenic bacteria in vegetable juice decreased by ~3 log CFU/mL at 5 h after treatment with the coliphage ECP4 (~10^8^ PFU/mL). Their results showed that the coliphage could be used to control *E. coli* O157:H7 in foods and eradicate biofilm formed by the pathogen. The application of different phages (O1, T1, T4, T5), independently or as a cocktail, was shown to be effective at controlling *E. coli* O157 on refrigerated beef [38]. The pathogen was most effectively controlled by the phage T5 at 4 and 22 °C; the viable count of *E. coli* O157 was reduced by 3.2 log_10_ CFU/cm^2^ following phage treatment at 4 °C for 144 h. 

Phages have a limited host range; this is a major disadvantage when using phages as biocontrol agents, which may be circumvented by using a phage cocktail [2]. Complete elimination of *E. coli* O157:H7 from beef meat surface following the application of anti-O157 phage cocktail (e4/1c, pp01, e11/2) was shown [2]. The lytic activity of a phage cocktail (ECP-100) consisting of three *Myoviridae* phages on three *E. coli* O157:H7 strains in experimentally contaminated ground beef, broccoli, spinach, and tomato was shown by Abuladze et al. [39]. They observed reductions in the range 94–100%. Tomat et al. [40] showed that phages DT1 and DT6 (when applied independently or as a cocktail) effectively reduced the viability of *E. coli* O157:H7 during milk fermentation, without compromising the starter culture performance. Tomat et al. [41] reported a complete inactivation (ca. 5–6 log CFU/mL) of O157:H7 STEC464 by using the phage cocktail (DT1+DT6, 1:1 ratio, ca. 10^9^ PFU/mL) on stainless steel coupons and glass coverslips at different multiplicities of infection (ca. 10^3^ and 10^7^) and temperatures (4 °C and 37 °C). They concluded that the lytic phages, either individually or as a cocktail, could be used for decreasing contamination on food-processing surfaces. Tomat et al. [42] demonstrated effective inactivation of pathogenic *E. coli* strains, including O157:H7 STEC, in meat and milk using the phage cocktail (which consisted of six phages: DT1-DT6) at abusive (37 °C) and room (24 °C) temperatures. A cocktail of phage M8AEC16 and phage M12BEC16 can be used to control *E. coli* O157:H7 effectively in pastirma (dried pressed meat) [43]. During the washing of produce, bacteriophages could be employed to remove foodborne pathogen contamination on vegetables and fruits. A cocktail of bacteriophages (L1, LL15, V9, C14s), which were specific for *E. coli* O157:H7, was shown to reduce the viability of *E. coli* O157:H7 significantly (99.99% reduction in 3 h) in an organic-rich environment [44]. In some circumstances, the limited host range of phages can be beneficial since the property of high specificity of phages may be used to develop a specific antimicrobial packaging. Such packaging materials kill the targeted pathogen only, leaving commensal bacteria unaffected. In a study, a cocktail of six lytic phages was added into whey protein concentrate (WPC) edible films and their stability, release, and antimicrobial efficacy against pathogenic *E. coli* strains were tested [45]. Their results showed that the phages were highly stable in WPC films, successfully released from the films, and confirmed to be very effective as a biocontrol tool. An additional advantage of a phage cocktail is that the emergence of phage-resistant mutant bacteria can be prevented by using an optimally composed phage cocktail [46]. As a downside, a combination of two or more different phages in preparation could lead to antagonism and recombination between phage members [47]. The use of bacteriophages with a wide host range could negate these disadvantages. Two novel rV5-like lytic bacteriophages (P206 and C203) were isolated from food samples. They exhibited a wide and uniform host range, including EHEC O157:H7. In experimentally inoculated minced meat, both of them effectively reduced viable populations of EHEC O157:H7 Sakai [48]. The potential of PS5 (a polyvalent phage), which is capable of simultaneously controlling *E. coli* O157:H7, *Salmonella* Typhimurium, and *Salmonella* Enteritidis, in inactivating these pathogens in foods was demonstrated by Duc et al. [47]. In broth, after incubation with the phage for 2 h at 4 °C and 24 °C, the viable counts of all the three bacteria were significantly decreased (>1.3 log CFU/mL) compared to the controls. Similarly, significant reductions were also observed in foods. Niu et al. [49] showed that the AKFV33 was capable of simultaneously decreasing viable counts of *Salmonella* and STEC in mixtures and the phage exhibited relatively higher lytic activity against serotypes O26:H11 and O157:H7. The results indicated that different zoonotic bacterial pathogens in food products can be concomitantly inhibited by using the phage without harming beneficial bacteria.

To control foodborne bacterial pathogens, the potential use of bacteriophages has been widely considered and some phage preparations have received generally recognized as safe (GRAS) status for use in foods [50]. However, some safety concerns associated with bacteriophage use include the carriage of virulence or antibiotic genes and their possible transfer (i.e., undesirable mobilization of genes), as aforementioned. These concerns could be nullified by the use of replication-deficient phages, which retain the host specificity and cell wall-degrading ability of the viable phage particle. Replication-deficient UV-treated phages were successfully used as an alternative to viable phages for controlling foodborne pathogens [50]. In a study, UV-treated phages were successfully applied to inactivate *E. coli* O157:H7 on the surface of raw and cooked meat and in milk [51]. Additional studies that have reported successful bacteriophage application for *E. coli* O157:H7 control in foods are given in Table 1.

### 2.2. Phage Stability

There are some challenges with regard to phage application in foods. In food matrices, a crucial factor in the effectiveness of phage treatment is the phage stability into the food; however, a constant phage titer is difficult to maintain since food systems are so complex [64,65]. Phage viability or phage attachment to the bacterial cell wall can be affected by any change in the structure of the phage. The response of phages to different foods is variable; factors such as properties of the food matrix, pH, and temperature were found implicated in this variability [66]. Phages lose their bioactivity in the presence of acidic compounds and enzymes. Antiphage activity of certain food compounds, which are originated from plant, animal, and other sources, such as dairy proteins, retinoids, alcohols, fatty acids, high sucrose and ionic (NaCl) concentrations, and cationic compounds, were discussed [65].

### 2.3. Stability Improvement Strategies

To enhance the stability of phages, a liposome encapsulation approach can be used. In a study, a chitosan-based edible film carrying liposome-encapsulated phages was developed [67]. When used as a packaging material for beef preservation, the prepared film displayed relatively high levels of bactericidal activity against *E. coli* O157:H7 and, consequently, the shelf life of the beef samples extended markedly, without any impact on sensory properties. Ramirez et al. [68] also showed that the survival of bacteriophages in hostile environments, such as UV light, extreme temperature, and extreme pH conditions, can be improved by microencapsulation. They showed that, compared with free phages, microencapsulated phages (ΦC119, ΦE142, ΦJLA23, ΦKP26) exhibited significantly (*p* < 0.05) higher stability upon exposure to UV irradiation, pH conditions between 3 to 7, and temperatures between at −80 °C and 70 °C. These results showed that, under stressful conditions, microencapsulation can provide additional stability to phage formulation and assure a more commercially appropriate formulation for controlling *E*. *coli* O157:H7. Lin et al. [58] improved the chemical stability of bacteriophages by incorporating them into a liposome system; the stability of the resultant phage-containing liposomes was further improved using a poly-L-lysine (PLL) coating. Anti-*E. coli* O157:H7 activity of bacteriophages encapsulated in PLL-coated liposomes was tested in model pork suspension; viable counts of *E. coli* O157:H7 were reduced by 2.44 log CFU/mL following 1 day of incubation. The result demonstrated that the encapsulated phages could extend the treatment time. An edible coating is a promising carrier of bacteriophages, whose release onto the food surface may control bacterial growth. A chitosan-based edible coating was used for phage VB_EcoMH2W stabilization without remarkable loss in its lytic activity over 1 week [69]. They also showed that an edible coating of chitosan incorporated with VB_EcoMH2W exhibited a significant antimicrobial activity (viable *E. coli* O157:H7 counts were reduced by 3 log cycles) compared to the control (coated without bacteriophages) after six days. The immobilization of bacteriophages on inert surfaces is an effective strategy for extending their range of application. A cocktail of *E. coli* O157:H7-specific phages immobilized on modified cellulose membranes was shown to be effective at controlling the growth of *E. coli* O157:H7 in raw and RTE meat under different packaging conditions and storage temperatures [70]. The immobilization method was based on the charge difference between the head and tail fibers of bacteriophages; their heads possess a net negative charge and the tail fibers possess a net positive charge. It is thus more likely that the heads would attach to positively charged surfaces, leaving the tails free to capture and lyse bacteria.

### 2.4. Sources of E. coli O157:H7-Specific Phages

Tomat et al. [54] isolated bacteriophages belonging to the *Myoviridae* family. These T-even-type coliphages were free from all the virulence factors tested, stable during storage, and exhibited lytic activity against several *E. coli* virotypes. Additionally, they could be used for STEC reduction in meat products. Two phages belonging to *Myoviridae* (BECP2) and *Podoviridae* (BECP6) were isolated from a sewage sample, and the application of a mixture of the phages decreased the growth of *E. coli* O157:H7 in commercial milk (viable counts reduced from 6 to 1 log CFU/mL) following incubation at 37 °C for 5 h [71]. In addition, the phage mixture was also shown to be effective for reducing *E. coli* in biofilm on a stainless steel coupon and a microtiter plate. They concluded that these phages could be used as potential biocontrol agents to simultaneously remove *E. coli* O157:H7 from biofilms and reduce *E. coli* O157:H7 in food. An alkali-resistant phage, namely, JN01, belonging to *Myoviridae* was isolated from wastewater and the phage displayed lytic activities against different strains of *E. coli*, including multi-drug resistant strains [4]. A significant reduction in viable counts of *E. coli* O157:H7 in milk and beef was achieved through the use of this phage (Table 1). An anti-*E. coli* O157:H7 phage, namely, FEC14, belonging to the *Kuttervirus* genus within the *Ackermannviridae* family was isolated from hospital sewage; endonuclease resistance of this phage makes it a potential candidate for controlling foodborne pathogens [72]. A virulent bacteriophage (phiC120) belonging to *Mosigvirus* genus and *Myoviridae* family was isolated from horse feces and it exhibited a broad lytic activity against several pathogenic *E. coli* O157:H7 strains [73]. Lee et al. [74] isolated a novel *E.*-*coli*-O157:H7-specific phage, namely, KFS-EC, belonging to the *Myoviridae* family from slaughterhouse wastewater and they suggested that the phage could be used as a biocontrol agent. A phage specific for *E. coli*, namely, vB_EcoM-ECP26, was isolated from a sewage sample; the phage belongs to the *Myoviridae* family and could be used for controlling foodborne STEC [62]. Bacteriophages specific for *E. coli* O157:H7, namely, vB_EcoS-P24 (belonging to the *Siphoviridae* family) and vB_EcoM-P23, vB_EcoM-P12, vB_EcoM-P34 and vB_EcoM-P13 (belonging to the *Myoviridae* family), were isolated from sewage and wastewater from a food plant [75]. They exhibited a wide host range, strong lytic activity, were stable at high temperature (up to 70 °C) and in a wide pH range (3–11 pH), and were found to be effective against *E. coli* O157:H7 under different storage conditions (5–37 °C). Broad-host-range phages, which belong to the *Siphoviridae* and *Podoviridae* family, were isolated from sewage water; a cocktail of these phages significantly reduced the growth of *E. coli* O157:H7 too, indicating their potential as a biocontrol agent [76]. A novel anti-*E. coli* O157:H7 phage, namely, F241, was isolated from a high salinity and acidic environment (i.e., an industrial cucumber fermentation plant); it exhibited lytic activity, high specificity, and tolerance to high salinity and low pH [28]. The phage (Φ241) was specific for O157 antigen and was capable of lysing different strains of *E. coli* O157:H7; therefore, it can potentially be used as a biocontrol agent against *E. coli* O157:H7 in foods. A novel *E*. *coli* O157:H7-specific phage, namely, HY01, belonging to the family *Myoviridae* was isolated from a swine fecal sample and was found to be stable under various pH and temperature conditions [56].

## 3. Plant-Derived Natural Compounds

Phytochemicals present in plants (e.g., flavonoids, alkaloids, tannins, steroids, and saponins) are partially responsible for their microbicidal activities [77]. Anti-*E. coli* O157:H7 activity of phytochemicals derived from medicinal plants, herbs, and spices was reported (please also refer to Table 2). 

Sakagami [81] showed that fruit extract of *Prunus mume* Sieb. et Zucc. can be used to inhibit the production of verotoxins by EHEC O157:H7. Hara-Kudo et al. [82] showed the bactericidal activity of green tea extract against *E. coli* O157:H7, where a complete inhibition of the growth of the pathogen was observed with the extract at a concentration of 500 μg/mL (minimum inhibitory concentration (MIC) was <250 μg/mL). In addition, the damage of cell membranes was observed with 1000 μg/mL of the extract. When foods are exposed to abusive temperatures (e.g., abusive refrigeration temperature of 8–10 °C), essential oils (e.g., tea tree and clove) could be used as potential bio-preservatives in order to control foodborne pathogens. Essential oils (EOs) extracted from tea tree and clove were used for controlling *E. coli* O157:H7 on minced cooked beef and blanched spinach [83]. Higher concentrations of both the EOs were required to control populations of the pathogen in minced cooked beef (4 times the MIC) and blanched spinach (3 times the MIC). The study also showed that the food composition, the oil concentration, and the storage temperature influenced the antimicrobial action of these EOs. However, the application of the oils at a higher concentration to these foods exerted a strong off-flavor. In another study, a significant (*p* < 0.05) decrease (1.90 and 1.65 log CFU/g) in the viability of *E. coli* O157:H7 on baby carrots and shredded lettuce, respectively, was observed upon the application of thyme oil (1.0 mL/L for 5 min) [84]. Harich et al. [85] showed the bactericidal activity of two natural bio-preservatives formulations against several pathogens, including *E. coli* O157:H7 EDL 933. The formulations were based on lemongrass EO/citrus extract/lactic acid (with a ratio of 0.01:0.1:1) for F2 and oregano/citrus extract/lactic acid (with a ratio of 0.01:0.1:1) for F6. Compared to the control and F6 formulations, the F2 formulation displayed a relatively strong inhibitory effect. In cranberries coated with F2 and F6 formulations, the total inhibition of the pathogen was noted on day 4 during storage at 4 °C. In terms of organoleptic properties, both formulations were acceptable. In contrast, no inhibitory effect of EOs of nutmeg and oregano against *E. coli* O157:H7 in ready-to-cook traditional Iranian barbecued chicken (TIBC) was shown, although the EOs showed an inhibitory effect against the pathogen in a broth system [86]. This could be due to the fact that the survival and growth of pathogenic bacteria in food products are known to depend on intrinsic factors (e.g., pH and acidity in the aqueous phase) and extrinsic factors (e.g., background flora, temperature). A detailed discussion on the role of EOs against pathogenic strains of *E. coli*, specifically O157:H7, and strategies to apply these compounds in food systems was recently given by Munekata et al. [87]. In a separate study, Ponce et al. [88] showed limited inhibitory effects of rosemary and oreganum oleoresins against inoculated *E. coli* O157:H7 in lettuce and carrot stored at 20 °C and 8 °C. Factors such as storage temperature and the type and concentration of oleoresins were shown to influence the microbicidal effects of oleoresins against *E. coli*. In general, phytochemicals exert their antibacterial activity through different mechanisms of action, including suppression of some virulence factors (inhibition of the activity of enzymes and toxins), damage to the bacterial membrane, and inhibition of bacterial biofilm formation [89]. Despite their potential antimicrobial properties, EOs have limited applications as food preservatives due to their intense aroma and toxicity issues. Some studies showed that EOs used as food preservatives may change the organoleptic properties of foodstuffs and their higher doses can produce severe toxicological responses [90,91]. Other drawbacks of phytochemicals as biopreservatives include their interaction with food ingredients, low water solubility, and they are often expensive [92].

## 4. Probiotics

As living microbial food ingredients, probiotics are known to exert a beneficial effect on human health [93]. Probiotic bacteria belonging to the genera *Lactobacillus* and *Bifidobacterium* are the most commonly studied. Lactic acid bacteria (LAB) strains isolated from foods are excellent candidates to form protective biofilms. Russo et al. [94] showed the antagonistic effect of the probiotic LAB *L. plantarum* B2 against *E. coli* O157:H7 on experimentally inoculated fresh-cut pineapples under refrigeration storage. Gómez et al. [95] showed that probiotic LAB biofilms, formed especially with *L. lactis* 368 (non-bacteriocin producer), *Lactobacillus sakei* MBSa1 (bacteriocin producer), and *Lactobacillus curvatus* MBSa3 (bacteriocin producer), exhibited the strongest bactericidal activity (>6 log reduction) against biofilms formed by *E. coli* O157:H7 and other pathogens through exclusion mechanisms. It is known that LAB produce biopreservatives, such as lactic acid, bacteriocins, and hydrogen peroxide, that can slow both spoilage and the growth of pathogenic bacteria [96]. Ayala et al. [97] used a systematic approach (a combination of phenotypic and genotypic assays) to identify safe and effective novel LAB probiotic strains that have the potential to exert antagonistic effect against different foodborne pathogens, including *E. coli* O157:H7. In their study, most of the isolated LAB probiotic strains (potential strain: *L. salivarius* L28) exerted an antagonistic effect against *E. coli* O157:H7 in different environments and matrices. Two probiotic strains of *Lactobacillus reuteri* and *Bifidobacterium longum* subsp. *infantis*, which can form biofilms on solid surfaces, were used for controlling the growth of food spoilage and pathogenic bacteria [98]. In the presence of the probiotic biofilm, cell loads of pathogenic bacteria were relatively low compared to its absence; the viable cell load of *E. coli* O157:H7 was significantly reduced (>1–2 log cycles). Alvarez et al. [99] showed the antagonistic effects of probiotics (*Lactobacillus rhamnosus* and *Bifidobacterium animalis* subsp. *lactis*) against *E. coli* O157:H7 inoculated on fresh-cut apple. Following storage for 8 days, compared with samples without probiotics, the viability of *E. coli* O157:H7 was significantly decreased (0.9 and 1.3 log) by the presence of *B. lactis* and *L. rhamnosus* in alginate-prebiotic coatings, respectively. In contrast, Bambace et al. [100] showed that viable populations of inoculated *E. coli* O157:H7 in blueberries were not significantly affected by the application of alginate-based prebiotic coating incorporating probiotic cultures, namely, *B. animalis* subsp. *lactis* CECT 8145 or *Lacticaseibacillus casei* CECT 9104. A previous study reported similar findings, wherein the probiotic strain *L. rhamnosus* CECT 8361 showed no antagonistic effect on the viability of inoculated *E. coli* O157:H7 (co-inoculated with *L. innocua*) in fresh blueberries during cold storage [101]. These contradictory results could be due to the fact that the success of probiotics as biopreservative agents primarily depends on the type of food matrix, type of foodborne pathogen, inoculum level, inoculation method, and contact possibilities between the probiotic and pathogen [100].

## 5. Other Antagonistic Bacteria

Janisiewicz et al. [102] successfully used the antagonistic bacterium *Pseudomonas syringae* (strain L-59-66), which is generally applied to control the postharvest decay of pome fruits caused by fungi, such as *Penicillium expansum* and *Botrytis cinerea,* to inhibit *E. coli* O157:H7 growth on wounded apple tissue. The mechanism of the antagonism is presumably due to competition for nutrients and space. Despite the low pH of apples and peaches, foodborne bacterial pathogens (inclusive of *E. coli* O157:H7) could continue to exist in acidic environments and cause public health issues. Antagonistic effect of a Gram-negative bacterium *Pseudomonas graminis*, which was isolated from whole ‘Golden Delicious’ apples, against *E. coli* O157:H7 on fresh-cut apple and peach has been shown by Alegre et al. [103]. The bacterium successfully inhibited the growth of *E. coli* O157:H7 on both fruits at refrigeration temperatures. Under co-inoculation conditions, *Pseudomonas* sp. M309 reduced viable counts of *E*. *coli* O157:H7 by 3.7 log units on lettuce disks following storage at 10 °C for 9 days [104]. According to their results, the microorganism can potentially be used as a biopreservative agent to control other foodborne pathogens. *E. coli* O157:H7 could internalize in and reside in sprout inner tissues and pose serious food safety challenges [105]. In a study, to control the internalization of *E. coli* O157:H7 505B (pathogen indicator) in mung bean sprouts, *Bacillus subtilis* antagonists isolated from lettuce stems and mung bean seeds were used [106].

The ratio of antagonist to pathogen greatly affects the effectiveness of biological intervention in the culture. Liao [107] showed that the inoculation of pepper disks with *Bacillus* YD1 and *Pseudomonas fluorescens* 2–79 at concentrations of 5–6 log CFU/disk was adequate to decrease the growth of selected human pathogens, including *E. coli* O157:H7, on pepper disks by 3–4 log units. Maximum reductions in viable pathogens were noted when antagonists were applied in higher numbers (10- to 100-fold high) than pathogens. To control the growth of *E. coli* O157:H7 on spinach, Olanya et al. [108] used *P. fluorescens* as a biocontrol agent. The average reduction in viability of the pathogen ranged between 0.5 and 2.1 log CFU/g of spinach, and they showed that the storage temperature significantly (*p* < 0.05) affected the efficiency of biocontrol since inhibitory effects were relatively higher at 15 °C than at other temperatures. Olanya et al. [109] reported that nutrient availability may impact *E. coli* O157:H7 growth in the presence of *P. fluorescens*. The suppressive levels were found to vary with pathogen-strain combinations, nutrient availability, and storage times. They showed that the survival of *E. coli* O157:H7 and *P. fluorescens* in co-cultures can be utilized to assess the biocontrol potential of *P. fluorescens* in reducing *E. coli* O157:H7 contamination on produce, especially during transitory temperature abuse conditions. Ponce et al. [88] showed biocontrol potential of endogenous microflora against *E. coli* O157:H7 in minimally processed carrot and lettuce. The native microflora exhibited bacteriostatic and bactericidal effects on the growth of the pathogen when the levels of inoculate were low and high, respectively. The competitive power of native microflora for physical space and nutrients could be an essential property for controlling the growth of the pathogen. Lim et al. [110] reported the antibacterial activity of *Pantoea agglomerans* R190, a Gram-negative bacterium, against human gastrointestinal pathogens, including *E. coli* O157:H7 and vegetable spoilage bacteria. A marine predatory bacterium, *Halobacteriovorax*, is known to modulate bacterial pathogens in shellfish. Richards et al. [111] isolated *Halobacteriovorax* strains that were predatory for bacterial pathogens, including *E. coli* O157:H7, which pose a potential threat to the safety of seafood. Olanya et al. [112] showed reductions in viable cell counts of *E. coli* O157:H7 on produce and a buffer in the presence of *Bdellovibrio bacteriovorus* 109 (Bb109), which is a predator of Gram-negative bacteria. In the buffer, the levels of inactivation of the pathogen ranged between 1.0 and 3.9 log CFU/mL, whereas on lettuce and carrot, 0.3–1.8 log reductions were noted. LAB strains (food isolates with biofilm-producing capacity) belonging to *Pediococcus pentosaceus*, *Pediococcus acidilactici*, and *Lactiplantibacillus plantarum* species were found to display the highest suppressive activities against EHEC O157:H7 at 10 °C [113]. Among the isolates, *L. plantarum* CRL 1075 was found to be effective against EHEC. Kim et al. [114] isolated competitive exclusion (CE) microorganisms from sprout seeds that were inhibitory to *E. coli* O157:H7. Among them, a biofilm-forming strain (T5) of *Paenibacillus polymyxa* showed strong antimicrobial activity. The results showed that biofilms formed by the strain T5 on stainless steel coupons effectively inhibited post-infection by *E. coli* O157:H7. Baker et al. [115] showed the antagonistic effect of soil bacterial isolates against *E. coli* O157:H7. Among the isolates, *Paenibacillus alvei* exhibited a potent bactericidal effect against *E. coli* O157:H7; viable cell counts of the pathogen were reduced by >3 log CFU/mL following incubation at 30 °C for 3 days.

## 6. Endolysins

Endolysins are bacteriophage-encoded enzymes. They can lyse the target bacteria without allowing for the development of resistant strains. Other merits of using endolysins as a biocontrol measure include specificity, they can act synergistically, ability to penetrate biofilms, environmentally friendly, and biodegradable [116]. Endolysin exerts its anti-bacterial effect by degrading peptidoglycan linkages and is regarded as more efficient than phages [117]. It was shown that endolysin (LysECP26) derived from rV5-like phage can lyse STEC O157:H7 [118]. However, in the case of Gram-negative bacteria, the access of bacteriophage endolysin to the peptidoglycan is prevented by the outer membrane and therefore, outer membrane permeabilizers are generally needed. LysECP26, in combination with outer membrane permeabilizers (EDTA and organic acids), could effectively control the pathogen, as LysECP26 may not traverse through the outer membrane. Another study showed the bactericidal activity of endolysin (LysF1) obtained from *E. coli* O157:H7 phage FAHEc1 against Gram-negative bacterial cells (chloroform-treated) [119]. Nevertheless, a protein engineering approach was used recently to modify endolysins in such a way that they can readily traverse the outer membrane and cause the lysis of Gram-negative bacteria cells thereafter [119,120]. Xu et al. [121] developed a novel phage lysin (PlyEc2) to kill pathogenic *E. coli* and other key Gram-negative pathogens on produce; a single dose (500 μg/mL, which is a dose corresponding to 111 μg of lysin/cm^2^ of treated leaf surface) of the lysin inactivated almost all *E. coli* O157:H7 cells (99.7%) on contaminated lettuce without negatively affecting the sensory characteristics of the treated leaves. 

However, there are immunogenicity and stability (as they are enzymes) concerns with regard to endolysin applications. Additional drawbacks include the restricted spectrum of activity and scalability challenges. To circumvent these problems, different strategies are being investigated. For instance, to enhance the thermal stability and antibacterial (enzymatic) activity of endolysins (e.g., LysF1) (since these characteristics are essential to optimize their implementation in various settings), a rational engineering approach such as site-specific mutagenesis could be used [119]. The encapsulation approach could be used for controlling the release of endolysins in response to various environmental cues in different food matrices, and the lytic spectrum of several endolysins can be extended by the creation of chimeric endolysins [122].

## 7. Bacteriocins

Bacteriocins are produced by both Gram-negative and Gram-positive bacteria living in a competitive polymicrobial environment. They are ribosomally synthetized peptides that target and kill or inhibit strains of closely related bacterial species [123]. The activity of bacteriocins can be attributed to their influence on the cytoplasmic membrane, resulting in the collapse of the proton motive force through the formation of pores in the phospholipid bilayer [92]. Bacteriocins have various desirable properties, such as they are natural compounds, generally recognized as safe (GRAS), do not cause any changes in the physicochemical properties of foods, and are degraded by proteases in the human gut [124]. Bacteriocins produced by *Enterococcus hirae* (Eh9) have been used for controlling microbial populations in lettuce seeds [125]. Cell-free supernatant of the Eh9 exhibited a significant biocontrol effect on exogenous *E. coli* O157:H7 ATCC 43895. Treatment with an E2-type colicin named Hu194 (a bacteriocin made by *E. coli*) had successfully decreased the viable counts of *E. coli* O157:H7 (ATCC43890, ATCC43895) from inoculated alfalfa seeds [126]. They showed approximately 5 log CFU mL^−1^ reductions following incubation with colicin (semi-crude) for 1 day and a successful elimination (5 log CFU g^−1^) of the strain 43890 from inoculated alfalfa seeds was noted following their soaking in a colicin suspension (10,000 AU/g). Öncül and Yıldırım [124] showed the bactericidal effect of bacteriocins, lactococcin BZ, and enterocin KP against *E. coli* O157:H7 in ultra-high temperature (UHT) milks when used independently or in combination at different concentrations (400–2500 AU/mL).

Yi et al. [127] investigated the inhibitory potential of an antimicrobial peptide zp37 (GIKAKIIIKIKK-NH_2_) against *E. coli* O157:H7 in sprouts. The peptide exhibited a MIC value of 16 μM on the pathogen with low toxicity. In sprouts, treatment with zp37 decreased the viability of the pathogen by 94.7% upon storage for 7 days. The mode of action studies indicated that the peptide caused weak membrane permeabilization and membrane depolarization, which consequently resulted in cellular deformation. Binding of the peptide (upon entering the cytoplasm) to the DNA of *E. coli* O157:H7 was observed, which resulted in DNA aggregation and precipitation. Corbalán et al. [128] showed *in vitro* antimicrobial activity of MccJ25(G12Y), which is a variant of the antimicrobial peptide microcin J25 (MccJ25), against *E. coli* O157:H7 NCTC 12900 in beef burgers and yogurt. In yogurt, the peptide (~63 μg/mL) reduced the viable counts of the pathogen by ~4 log_10_ CFU/mL on day 5, and on the beef burgers, the peptide (~63 μg/g) decreased the number of viable *E. coli* O157:H7 by ~3 log_10_ CFU/g on day 10. In a study, a synergistic antimicrobial effect of lysozyme with the ovine peptide SMAP29 (RGLRRLGRKIAHGVKKYGPTVLRIIRIA-NH_2_), lactoferrin, and polymyxin B against *E. coli* O157:H7 was shown [129]. The results also showed synergy between SMAP29 and lactoferrin against this host. The drawbacks of bacteriocins as biopreservatives include limited diffusion in solid matrices, narrow activity spectrum, spontaneous loss of bacteriocinogenicity, interaction with food ingredients, inactivation through proteolytic enzymes, and bacteriocin-resistant bacteria [92].

## 8. Bio-Enzymes

The efficacy of extracellular polymeric substances (EPS)-degrading enzymes, namely deoxyribonuclease I (DNase I), cellulase, and proteinase K, for controlling the formation of biofilm or eradication of pre-existing *E. coli* O157:H7 biofilms was studied [130]. Biofilm formation by the pathogen was significantly inhibited in the presence of cellulase or proteinase K; in particular, biofilm inhibition was synergistically improved by proteinase K combined with NaClO treatment. A significantly higher synergistic inactivation of *E. coli* O157:H7 was noted following sequential treatment with proteinase K, cellulase, and NaClO.

## 9. Hurdle Technology

Hurdle technology is the successive or simultaneous application of selected food processing techniques (two or more) for enhancing food safety and quality using lower individual treatment intensities [131]. Hurdle technology disrupts homeostasis causing metabolic exhaustion and cell death. Additive or non-additive (synergistic/antagonistic) effects could result from this combination approach. Phages can exhibit a synergistic effect with other antimicrobial agents. In a study, the sequential treatment of cold nitrogen plasma (CNP) and phages specific to *E. coli* O157:H7 exerted a superior anti-biofilm effect [132]. Cui et al. [133] showed the synergistic effect of clove oil and CNP against biofilms formed by *E. coli* EHEC O157:H7 on lettuce. Some published reports on anti-*E. coli* O157:H7 effects of various combinations of biocontrol agents and a biocontrol agent together with another non-thermal antimicrobial agent (including phage cocktail BEC8 + *trans*-cinnamaldehyde, green tea extract + RF cold plasma, PEF + carvacrol, garlic essential oil + allyl isothiocyanate (AITC), AITC + o-coumaric acid) are given in Table 3.

## 10. Conclusions

In general, a highly lytic single bacteriophage would decrease complications arising from competitive interference, as well as expenses for the generation and purification of phages. However, to control a broad range of pathogens, phage cocktails would be ideal as they contain sufficient diversity of phages. In hurdle technology, the synergism between biocontrol agents belonging to two or more different groups or biocontrol agents, together with another antimicrobial agent (e.g., high hydrostatic pressure, microwave irradiation, pulsed ohmic heating, and pulsed light) may reduce the dose required to achieve a significant reduction; however, further studies are needed to assess their safety. On the other hand, hurdle technology could compromise phage activity since emerging technologies can interfere with phage stability. An undesirable side effect of phage therapy is the release of endotoxins following lytic phage-mediated rapid lysis of bacteria. To mitigate this issue, the application of non-lytic phages, which encode proteins that are lethal to their host without lysis and endotoxin release, was suggested. To control *E. coli* O157:H7, a genetically modified non-replicating M13-derived phage that expresses a lethal catabolite gene activator protein (CAP) was constructed [139]. The constructed phage successfully controlled *E. coli* O157:H7 in contaminated milk. Their results indicated that genetically engineered non-lytic phages could be used as bactericidal agents.

## Figures and Tables

**Table 1:** List of some studies that reported successful application of phages for *E. coli* O157:H7 control in foods.

Bacteriophage	Target Strain	Result	Reference
Lytic bacteriophage Av-08	*E. coli* O157:H7 on chicken skin surface	4.9 log_10_ CFU/4 cm^2^ reduction	[52]
Phage phiJLA23	*E. coli* O157:H7	High lytic activity	[53]
Phages DT1 and DT6, either alone or mixed in a cocktail (10^7^–10^8^ PFU/mL)	*E. coli* O157:H7 STEC464 (final concentration approx. 5 × 10^2^–5 × 10^3^ CFU/mL)	The cocktail rapidly and completely inactivated the pathogen	[40]
Phage DT6 (1.4 × 10^10^ PFU/mL) Phage cocktail (DT1 and DT6 in equal proportions)	*E. coli* O157:H7 STEC 464	Viable cell reduction of 1.15 log after 6 h The phage cocktail reduced viable counts by up to 2.58 log at 6 h and 2.20 log at 24 h	[54]
Phage FAHEc1 at 10^7^ PFU/mL	*E. coli* O157:H7 in broth *in vitro* at 5 °C	A reduction of 4 log_10_	[32]
Phage FAHEc1 at 3.2 × 10^7^ PFU/4 cm^2^	*E. coli* O157:H7 on pieces of sliced beef at 37 °C	A reduction of >2.7 log_10_	[32]
Phage FAHEc1 (10^7^ PFU/cm^2^ UV-treated phages (pre-UV treatment titer))	*E. coli* O157:H7 on raw meat (10^6^ CFU/cm^2^)	Viable counts were reduced by 1–2 log_10_ CFU/cm^2^	[51]
Coliphage ECP4 (with 8 log PFU/mL)	*E. coli* O157:H7 on cabbage (7–8 log CFU/g)	Complete reduction at 3 h after treatment with the phage	[37]
Phage M8AEC16	*E. coli* O157:H7 in raw meatballs	Reduction of viable counts by 0.69–2.09 log CFU/g in the first 5 h of replica trials	[34]
*E. coli* phage OSY-SP (10^8^ PFU/mL)	*E. coli* O157:H7 EDL933 on green peppers *E. coli* O157:H7 B6–914 on baby spinach	Reduced by 2.4–3.0 log CFU/g (5 min rinse) Reduced by 3.4–3.5 log CFU/g (2 min rinse)	[55]
Bacteriophage HY01	*E*. *coli* O157:H7 strains (ATCC 43890 and ATCC 43895)	>2 log reductions during the first 2 h of incubation	[56]
Bacteriophage phiE142	Multidrug-resistant *E. coli* O157:H7 strains	Lysis and wide host range	[57]
Phage M8AEC16 (at 5.0 log initial MOI)	*E. coli* O157:H7 strain ATCC 43895 on RTE Italian salads (4.3 log CFU/g initial count)	2.7 log CFU/g reduction in 5 h incubation at 22 °C	[35]
*E. coli* O157:H7 phage (1.5 × 10^9^ PFU/mL)	*E. coli* EHEC O157:H7 CICC 21530 on nutrient agar plates (approx. 10^5^–10^6^ CFU/mL)	~3.88 log CFU/mL reductions after 8 h of treatment and no visible colonies observed when reacting time extended to 12 h	[58]
Phage PE37	STEC O157:H7 in broth Raw beef artificially contaminated with STEC O157:H7	2.6 and 4.9 log CFU/mL reductions following incubation for 6 h at 8 and 25 °C, respectively 0.9 and 2.3 log CFU/piece reductions following storage for 24 h at 8 and 25 °C, respectively	[59]
Alkali-resistant phage JN01 (10^9^ PFU/mL)	*E. coli* O157:H7 in tryptic soy broth (10^3^ CFU/mL) *E. coli* O157:H7 in UHT milk (2.3 log_10_ CFU/mL) *E. coli* O157:H7 on beef samples (4.5 log_10_ CFU/cm^2^)	A complete inhibition within 48 h Undetectable within 1–3 days of storage at 4 °C A reduction of >2 log_10_ CFU/cm^2^ during storage at 4 °C	[4]
Bacteriophage ECPS-6 (MOI: 5 at 25 °C) (MOI: 50 at 25 °C)	*E. coli* O157:H7 A-1 and A-2 in filtered raw milk (1 × 10^5^ CFU × mL^−1^) *E. coli* O157:H7 A-2 in unfiltered raw milk (1 × 10^5^ CFU × mL^−1^)	2.97 log_10_ CFU × mL^−1^ and 4.1 log_10_ CFU × mL^−1^ reductions after 24 h The contamination level was below the detection limit after 6 h of incubation	[60]
EcoShield PX™, a cocktail of lytic bacteriophages (5 × 10^6^ and 1 × 10^7^ PFU/g) (ca. 1 × 10^6^ PFU/g)	*E. coli* O157:H7 (ca. 3.0 log CFU/g) in beef chuck roast, ground beef, cooked chicken, chicken breast, cheese, salmon, romaine lettuce, and cantaloupe *E. coli* O157:H7 (ca. 1 to 10 CFU/10 g) on beef chuck roast samples	Significant reductions (*p* < 0.05), up to 97% in all foods Significantly reduced (*p* < 0.05) by ≥80%	[61]
Phage vB_EcoM-ECP26 (10^6^ PFU/mL)	*E. coli* O157:H7 NCCP 13930 on romaine lettuce at 4 °C	Viable counts reduced to undetectable levels in 5 days	[62]
Phage FP43	A mixed biofilm of EHEC O157:H7 and *E. coli* O91:H−	Decreased the formation of biofilm by 82.4% Following 6 h of incubation, viable counts of total cells in the biofilm and *E. coli* O157:H7 were reduced by 2.85 and 2.76 log, respectively	[63]

**Table 2:** Anti-*E. coli* O157:H7 activity of certain phytochemicals.

Phytochemical	Target Strain	Result	Reference
Propolis extract (2% and 5% concentrations)	*E. coli* O157:H7 in apple juice	Viable counts were reduced to undetectable levels within 24 h	[78]
Aqueous bark extracts of *Quillaja saponaria*	*E. coli* O157:H7	>6.0 log CFU reductions at 37 °C within 1 h	[79]
Liposomal encapsulated (0.5% *w*/*w*) thyme extract	Inoculated *E. coli* O157:H7 in silver carp mince	Viable counts of the pathogen were reduced below the acceptable level (<2 log CFU/g) from day 9 to day 15 of storage	[80]

**Table 3:** Synergistic effects of combinations of biocontrol agents or a biocontrol agent together with another antimicrobial agent against *E. coli* O157:H7.

Target Strain	Synergistic Method	Result of the Study	Reference
*E. coli* EHEC O157:H7 CICC 21530	The sequential treatment of cold nitrogen plasma (400 W, 2 min) and *E. coli* O157:H7 phages (5%, 30 min)	Viable population of biofilms of the pathogen was decreased by 2 log CFU/cm^2^ following individual treatment, while the sequential treatment decreased viable count by 5.71 log CFU/cm^2^	[132]
*E. coli* EHEC O157:H7 CICC 21530 biofilms on lettuce	The combined treatment of clove oil (4 mg/mL, 30 min) and cold nitrogen plasma (400 W, 3 min)	5.48 log CFU/cm^2^ reduction with no effect on the appearance quality of lettuce	[133]
*E. coli* O157:H7 on leafy green vegetables (lettuce and spinach)	A cocktail of phages (BEC8, approx. 10^6^ PFU/leaf) and the essential oil *trans*-cinnameldehyde (TC, 0.5% *v/v*)	Upon treatment of both leaves with BEC8 or TC individually, no survivors were detected after 24 h at 23 and 37 °C at low levels of inoculum; when the two treatments were combined, complete inactivation (5 log CFU/leaf reduction) occurred within 10 min at all inoculum levels and temperatures on both leaves	[134]
*E. coli* on the surface of inoculated fresh-cut dragon fruit	The combined treatment of green tea extract (at tea: water; g/mL, ratio of 5%) and radio frequency (RF) cold plasma (40 W)	A complete inhibition (~5 log CFU/g)	[135]
*E. coli* O157:H7 in juices	Simultaneous application of pulsed electric fields (PEFs, 30 kV/cm) and 1.3 mM of carvacrol (a major component of certain essential oils)	5 log_10_ cycles of inactivation of *E. coli* O157:H7 in less than 50 pulses	[136]
*E. coli* O157:H7 in pork sausage	Garlic essential oil (GO) and allyl isothiocyanate (AITC) 125 ppm GO + 250 ppm AITC 250 ppm GO + 250 ppm AITC	1.01–1.87 log CFU/g reduction of the initial count after 20 days at 6 °C storage	[137]
*E. coli* O157:H7 in dry-cured sausage	AITC + o-coumaric acid (CA) at 20 × FIC (fractional inhibitory concentration)	Reduced by ≥5 log CFU/g after 21 days	[138]

## Data Availability

Not Applicable.
